# A Networked Method for Multi-Evidence-Based Information Fusion

**DOI:** 10.3390/e25010069

**Published:** 2022-12-30

**Authors:** Qian Liang, Zhongxin Liu, Zengqiang Chen

**Affiliations:** College of Artificial Intelligence, Nankai University, No. 38 Tongyan Road, Jinnan District, Tianjin 300350, China

**Keywords:** data fusion, Dempster–Shafer theory, evidential conflict, sensor malfunction, evidence interaction

## Abstract

Dempster–Shafer evidence theory is an effective way to solve multi-sensor data fusion problems. After developing many improved combination rules, Dempster–Shafer evidence theory can also yield excellent results when fusing highly conflicting evidence. However, these approaches still have deficiencies if the conflicting evidence is due to sensor malfunction. This work presents a combination method by integrating information interaction graph and Dempster–Shafer evidence theory; thus, the multiple evidence fusion process is expressed as a network. In particular, the credibility of each piece of evidence is obtained by measuring the distance between the evidence first. After that, the credibility of the evidence is evaluated, keeping the unreliable evidence out of the information interaction network. With the fusion of connected evidence, the accuracy of the fusion result is improved. Finally, application results show that the presented method is effective.

## 1. Introduction

With the advancement of science and technology in recent years, information analysis has gained popularity as a research topic. Applying multi-sensor data fusion techniques will enable the creation of the consolidated view within this process. Therefore, by utilizing multi-sensor data fusion technology in various decision-making applications, such as risk analysis [1,2], fault diagnosis [3,4], health prognosis [5], image processing [6], target tracking [7] and so on, the performance of the system is significantly improved. Meanwhile, researchers have long been troubled by the imprecision and uncertainty brought on by poor weather, old sensors, and lack of energy supply. Therefore, a fusion mechanism must be created in order to reduce this kind of ambiguity and imprecision.

Numerous theories have been put forth up to this point in order to model and handle uncertain and imprecise information, such as rough sets theory [8,9], fuzzy sets theory [10,11,12], evidence theory [13,14,15], Z numbers theory [16,17] and D numbers theory [18,19]. These methods, which have been widely used in a variety of fields depending on their needs, concentrate on various aspects. In particular, because Dempster–Shafer evidence theory (DST) can accept incomplete data and assign evidence to multiple hypotheses, there is less of a need for prior probabilities. Once the fusion result is obtained, the mass of belief can be transferred onto single hypotheses by pignistic transformation [20] or DSmP [21]. It has thus been favored for its adaptability and effectiveness in modeling uncertainty and imprecision.

DST undoubtedly has some drawbacks. Different degrees of conflicts are generated by the sensors due to different types, tasks, and precision. However, it is usually less likely to produce utterly different evidence if the sensor works normally. In most cases, the evidence obtained is relevant to a certain extent and contains complementary information. However, when strongly conflicting information due to malfunction is combined, the results might be unexpected [22]. There are primarily two ways to approach this problem after extensive research over many years. The first kind is to modify Dempster’s combination rule, for example, Yager’s combination rule [23], Dubois and Prade’s disjunctive combination rule [24], Smets’ unnormalized combination rule [25] and Martin and Osswald’s PCR6 rule [26,27]. Numerous studies are inclined to pretreat the body of evidence to handle the conflict evidence fusion problem because it is possible for some strong properties to be destroyed by modifying the combination rule. Specifically, Murphy’s simple average approach of the bodies of evidence [28], Deng et al.’s weighted average of the mass functions based on the evidence distance [29], Yuan et al.’s entropy-based method [30], Yang et al.’s open Deng entropy based method [31], Xiao’s prospect-theory-based method [32] and belief divergence measure based method [33] all focus on the second way. All this research has presented effective solutions for fusing conflicting evidence. Still, if conflicting evidence is due to sensor malfunction, the modification would not work as expected, and the preprocess also fails to remove the influence of fault information.

As a complex combination problem involving multiple sensors, it makes sense to think about a connection between multi-sensor data fusion and multi-agent systems. Artificial intelligence has long been interested in multi-agent systems, which have solved a number of issues that were too complex for a single agent [34]. Communication topology-based information exchange is crucial for a well-coordinated multi-agent system. Therefore, it is worthwhile to try to enhance communication in order to increase the accuracy of data fusion. Multi-agent systems will also be more successful in achieving this goal as the fusion result becomes more accurate [35]. However, different from multi-agent systems, since the reliability of information obtained by each sensor is different when sensing different environments, and where or when the malfunction will happen is unknown, there is a contradiction between presetting topology and obtaining higher information accuracy. That is to say, in order to obtain higher fusion accuracy, switching topology, or how establishing the most effective topology is a problem that must be taken into account. It is thus more reasonable to make a connection based on the reliability of the evidence.

As a result of the discussion above, a networked approach is used in this paper by connecting graph theory and evidence theory. A newly developed credibility test rule is used to weed out implausible evidence based on the credibility degree of the evidence. The effect of fault information is therefore eliminated due to the faulty sensor’s information being cut off from the communication network. It is worth noting that we assume the evidence used for fusion is already output by each sensor, but how the sensor obtains the output is not within the scope of this manuscript.

The main contributions of this study are as follows: (1) A networked information fusion method is proposed to solve the conflict evidence fusion problem. Inspired by the knowledge of graph theory in multi-agent systems, the proposed method takes the information interaction between evidence into account, which makes multi-data fusion problems closer to multi-agent systems. (2) Rather than exclude the evidence with the lowest credibility simply, a credibility degree test rule is defined for the purpose of checking whether the evidence contains enough valid information to participate in the fusion. In this way, accidents where credible evidence is omitted due to the order of credibility are avoided as much as possible. (3) According to the isolated node mechanism of the proposed method, the highly conflicting evidence provided by the faulty sensor can be distinguished as an isolated node. Furthermore, since the isolated node is not involved in the fusion process, the negative impact caused by the fault information can be reduced effectively.

The rest of this paper is organized as follows. Section 2 introduces the preliminaries of this paper briefly. The evidence interaction is explained to point out the direction for the fusion of evidence in Section 3. A graph theory and evidence theory based multi-sensor data fusion method is proposed in Section 4. In Section 5, a numerical example is illustrated to verify the effectiveness of the proposed method. The conclusion is summarized in Section 6.

## 2. Preliminaries

### 2.1. Dempster–Shafer Evidence Theory

DST was named by Dempster [13] and Shafer [14], who firstly proposed and developed the theory. To model the uncertainty and aggregate different information, it is necessary to comprehend the following basic concepts in DST.

**Definition 1**  ((Frame of discernment (FOD)) [33])**.** *Assume U is a set consisting of mutually exclusive and collectively exhaustive events; the set U is called a frame of discernment, which indicted by*

(1) $$U=\{E_{1},E_{2},\ldots ,E_{N}\}$$

*$2^{U}$ indicates the power set of U and contains all propositions, where*

(2) $$2^{U}=\left\{⌀,\left\{E_{1}\right\},\left\{E_{1},E_{2}\right\},\ldots ,U\right\}$$

*and ⌀ is an empty set.*

**Definition 2**  ((Mass function) [33])**.** *A FOD U is based on the concept of mass function, which is a mapping m(·) from $2^{U}$ to [0,1], satisfying the following condition:*

(3) $$m\left(⌀\right)=0,\sum_{A\in 2^{U}}m\left(A\right)=1$$

*In DST, mass function is also known as basic belief assignment (BBA). If $A\in 2^{U}$ verifies m(A)≥0, A will be called as a focal element, and the union of all of focal elements form the core of the mass function.*

**Definition 3**  ((Belief function) [33])**.** *The belief function and plausibility function of a proposition A∈U is defined as*

(4) $$Bel\left(A\right)=\sum_{B\subseteq A}m\left(B\right)$$

(5) $$Pl\left(A\right)=1-Bl\left(\bar{A}\right)=\sum_{B\cap A\neq ⌀}m\left(B\right)$$

*where $\bar{A}=U-A$ and Bel(A)≤Pl(A) are always satisfied.*

**Definition 4**  ((Dempster’s combination rule) [33])**.** *Dempster’s combination rule uses the orthogonal sum of two BBAs. Assume two independent BBAs $m_{1}\left(\cdot \right)$ and $m_{2}\left(\cdot \right)$ are on the FOD U, their combination $m\left(\cdot \right)=m_{1}\left(\cdot \right)⊕m_{2}\left(\cdot \right)$ is expressed as follows:*

(6) $$m\left(A\right)=\left\{\begin{array}{cc} \frac{1}{1-K}\sum_{B\cap C=A}m_{1}\left(B\right)m_{2}\left(C\right), & \;A\neq ⌀\\ 0, & \;A=⌀ \end{array}\right.$$

(7) $$K=\sum_{B\cap C=⌀}m_{1}\left(B\right)m_{2}\left(C\right)$$

*where B and C are also propositions in $2^{U}$, and K represents the conflict between two BBAs.*

### 2.2. Graph Theory

Graphs have been used to model information exchange between agents in cooperative multi-agent systems for years [36,37]. A graph consists of a pair (N,E), where N is a finite nonempty set of nodes, and E∈N×N is a set of ordered pairs of nodes, which are called edges. The pairs of nodes in an undirected graph are unordered. A directed path is a sequence of ordered edges of the form $\left(v_{i},v_{j}\right)$, where $v_{i},v_{j}\in N$ in a digraph. Nodes are connected if there is at least one path between two nodes. On the contrary, isolated nodes are not connected to any other nodes.

### 2.3. The Distance Measure between Basic Belief Assignments

The measure of similarity between BBAs is a natural consideration. The distance measure between BBAs can tell us whether two pieces of evidence are close or distant. In this regard, Jousselme’s distance [38] is a appropriate approach.

**Definition 5**  ((Jousselme Distance) [38])**.** *The distance between $m_{i}\left(\cdot \right)$ and $m_{j}\left(\cdot \right)$ is defined as:*

(8) $$d_{ij}=\sqrt{\frac{1}{2}{\left[m_{i}\left(\cdot \right)-m_{j}\left(\cdot \right)\right]}^{T}\underline{D}\left[m_{i}\left(\cdot \right)-m_{j}\left(\cdot \right)\right]}$$

*where $\underline{D}$ is an $2^{N}\times 2^{N}$ matrix whose elements are*

(9) $$\underline{D}\left(A,B\right)=\frac{\left|A\cap B\right|}{\left|A\cup B\right|},A,B\subseteq 2^{U}$$

*|A| is the cardinality of A.*

## 3. Evidence Interaction

How to deal with the conflict between evidence in DST has been a problem bothering researchers for a long time. Jousselme’s distance provides an easy and effective way to compare evidence. For instance, a small distance implies the support from other evidence, which means this evidence should play a more important role in fusion. Conversely, a large distance means this evidence is not supported by other evidence, that is to say, an insignificant role should be distributed to this evidence. However, only the similarity measure is still not enough to generate an accurate fusion result.

In order to better utilize the accurate information provided by the evidence, the credibility of the evidence is necessary. Furthermore, the case where the sensor failure happened is unavoidable. How can we know a sensor is malfunctioning or functioning normally? Assuming a sensor does fail, how can we know how badly it has failed? Can the information provided by the faulty sensor still be used for fusion? To answer these questions, the graph theory is leveraged to describe the interaction between the evidence in this section. According to the Jousselme distance, the credibility degree is obtained to measure the relative importance of evidence. What is more, the credibility is utilized to determine whether evidence obtained by a faulty sensor should be involved in the fusion process or not.

**Definition 6**  ((Divergence Measure) [32])**.** *Through Jousselme’s distance, the divergence measure matrix, denoted as DMM, can be obtained:*

(10) $$DMM=\left(\begin{array}{ccccc} 0 & \cdots & d_{1i} & \cdots & d_{1k}\\ \vdots & \cdots & \vdots & \cdots & \vdots \\ d_{i1} & \cdots & 0 & \cdots & d_{ik}\\ \vdots & \cdots & \vdots & \cdots & \vdots \\ d_{k1} & \cdots & d_{ki} & \cdots & 0 \end{array}\right)$$

*where $d_{ii}=0\left(1\leq i\leq k\right)$ represents the BBA $m_{i}$ is identical to itself.*

**Definition 7**  ((Support Degree) [33])**.** *Let $m_{1},m_{2},\cdots ,m_{i},\cdots ,m_{k}$ be the BBAs on the FOD U, we denote the support degree of BBA $m_{i}$ from other BBAs as $Sup_{i}$, which is defined as:*

(11) $$Sup_{i}=\frac{k-1}{\sum_{j=1}^{k}d_{ij}},j\neq i$$

*The support degree is the reciprocal of the average distance between $m_{i}$ and the other BBAs.*

**Definition 8**  ((Credibility Degree) [33])**.** *The credibility degree of BBA $m_{i}\left(1\leq i\leq k\right)$ on FOD U, denoted as $Crd_{i}$, is defined as*

(12) $$Crd_{i}=\frac{Sup_{i}}{\sum_{j=1}^{k}Sup_{j}}$$

**Definition 9**  (Evidence Connection). *Let a fully connected undirected graph G represent the initial topology of BBAs; the element of the initial adjacency matrix A is defined as*

(13) $$a_{ij}=1,\left(i\neq j\right);a_{ij}=0,\left(i=j\right)$$

*To determine whether BBA $m_{i}\left(\cdot \right)$ obtained by a faulty sensor is reliable or not, the credibility should be tested. If the following condition holds, $m_{i}\left(\cdot \right)$ is considered as a unreliable evidence.*

(14) $$\frac{\bar{Crd}-Crd_{i}}{Crd_{i}}\geq 1$$

*Once the BBA $m_{i}$ is considered as unreliable, the connection between $m_{i}$ and other BBAs will be disconnected. Correspondingly, $a_{ij}\left(j\neq i\right)$ and $a_{ji}\left(j\neq i\right)$ will be replaced by 0. The modified graph will represent the interaction between evidence, and the last BBAs will be fused following this information flow, which is detailed in the next section.*

## 4. The Proposed Method

**Figure 1:**
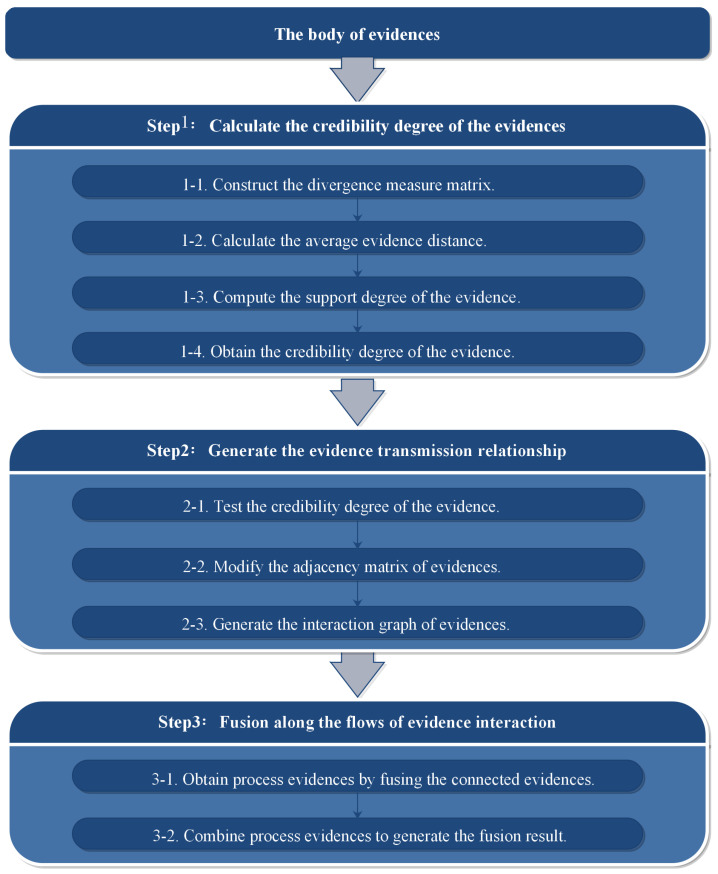
The flowchart of the proposed method.

The previous descriptions provided sufficient theoretical preparation for the fusion problem considered in this paper. Thus, a new networked method is presented to process the fusion problems in this section, which contains the following procedures. Firstly, the credibility degree of evidence can be measured via Jousselme’s distance. Secondly, by means of the relative significance hidden in the credibility, the transmission relationship between evidence can be generated, in which the impact of faulty information is reduced. Lastly, following the evidence interaction flows, the fusion result can be obtained through Dempster’s combination rules by two rounds. The procedures of the proposed approach are depicted in Figure 1.

### 4.1. Calculate the Credibility Degree of the Evidence

As mentioned above, it is necessary to take advantage of the signals provided by credibility degree. Hence, the credibility degree of the evidence will be obtained via the following steps.

Step 1-1: By mean of Jousselme’s distance, the divergence measure matrix DMM can be constructed as (10).
Step 1-2: For $m_{i}$, the average distance $AD_{i}$ from other evidence can be calculated by
  (15) $$AD_{i}=\frac{\sum_{j=1,j\neq i}^{k}d_{ij}}{k-1},1\leq i\leq k;1\leq j\leq k$$
Step 1-3: The support degree $Sup_{i}$ of $m_{i}$ is defined as
  (16) $$Sup_{i}=\frac{1}{AD_{i}},1\leq i\leq k$$
Step 1-4: The credibility degree $Crd_{i}$ of $m_{i}$ is calculated by
  (17) $$Crd_{i}=\frac{Sup_{i}}{\sum_{j=1}^{k}Sup_{j}},1\leq i\leq k$$

### 4.2. Generate the Evidence Transmission Relationship

Now, we obtained the credibility of the evidence. According to the conception of the proposed method, the transmission relationship between evidence can be established as follows.

Step 2-1: For each evidence, the credibility degree is tested by (14).
Step 2-2: For evidence $m_{i}$ satisfying (14), replace $a_{ij}\left(j\neq i\right)$ and $a_{ji}\left(j\neq i\right)$ in (13) by 0.
Step 2-3: In accordance with the modified adjacency matrix $A^{\prime }$, the interaction graph $G^{\prime }$ between evidence is constructed.

### 4.3. Fusion along the Flows of Evidence Interaction

The interaction graph between the evidence is now generated; it is time to fuse the evidence and obtain the combination result via the steps below.

Step 3-1: For each evidence $m_{i}$ in connected part of the interaction graph $G^{\prime }$, fuse evidence $m_{i}$ and evidence connected to $m_{i}$ directly via the Dempster’s combination rule (6); the fusion result is represented as process evidence $\underline{m_{i}}$.
Step 3-2: All process evidence obtained in the previous step are fused via Dempster’s combination rule (6). Hence, the final combination result is obtained.

As explained above, the proposed method still belongs to the second scheme introduced in Section 1. In the preprocess, credibility is calculated as a standard to measure the relative importance of evidence like many previous works. However, different from the previous weighting operations, the weighting is represented from another perspective in the form of the evidence interaction graph in the proposed method. The reason for utilizing the evidence interaction graph is that we attend to express and process the multi-data fusion problems in the form of networks. Furthermore, the unreliable evidence is weeded out after the credibility test, which prevents the false evidence from adversely affecting the outcome. As described in this section, the proposed method is theoretically feasible. To verify the feasibility of the proposed method in applications, examples are given in the next section.

## 5. Example Illustration

In order to demonstrate the effectiveness of the proposed method, two application cases from [33,39] are illustrated in this section.

### 5.1. Case 1

- Problem statement

Consider a target recognition problem associated with sensor reports collected from five different types of sensors. It should be noted that this is a fictitious example, and target A is supposed as the real target. These sensor reports, which are modeled as the BBAs, are given in Table 1 from [33], where the FOD *U* that consists of three potential objects is given by U=A,B,C.

- The fusion approach

**Figure 2:**
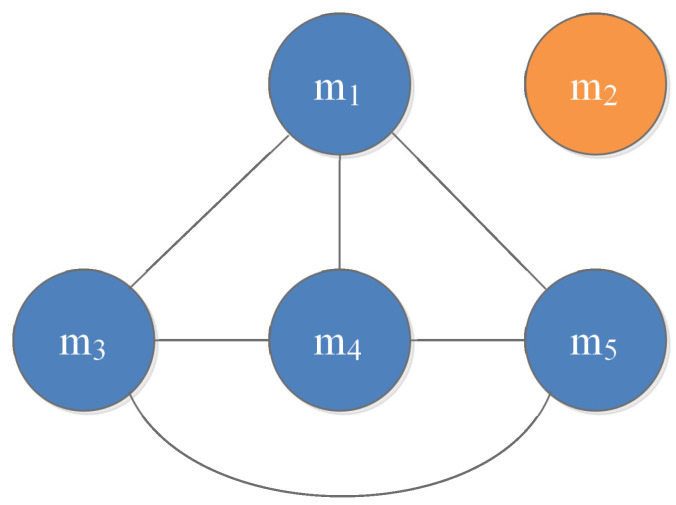
The interaction graph generated by the proposed method.

Step 1-1: Construct the divergence measure matrix $DMM={\left(d_{ij}\right)}_{k\times k}$ as follows:
  $$DMM=\left(\begin{array}{ccccc} 0 & 0.5386 & 0.3495 & 0.3257 & 0.3311\\ 0.5386 & 0 & 0.8142 & 0.7850 & 0.7906\\ 0.3495 & 0.8142 & 0 & 0.0300 & 0.0374\\ 0.3257 & 0.7850 & 0.0300 & 0 & 0.0354\\ 0.3311 & 0.7906 & 0.0374 & 0.0354 & 0 \end{array}\right)$$
Step 1-2: Obtain the average evidence distance $AD_{i}$ of $m_{i}$ as:
  $$\begin{array}{cc} \; & AD_{1}=0.3862\;AD_{2}=0.7321\\ \; & AD_{3}=0.3078\;AD_{4}=0.2940\\ \; & AD_{5}=0.2986 \end{array}$$
Step 1-3: Calculate the support degree of $m_{i}$ as:
  $$\begin{array}{cc} \; & Sup_{1}=2.5892\;Sup_{2}=1.3659\\ \; & Sup_{3}=3.2491\;Sup_{4}=3.4011\\ \; & Sup_{5}=3.3490 \end{array}$$
Step 1-4: Compute the credibility degree of $m_{i}$ as:
  $$\begin{array}{cc} \; & Crd_{1}=0.1855\;Crd_{2}=0.0979\\ \; & Crd_{3}=0.2328\;Crd_{4}=0.2437\\ \; & Crd_{5}=0.2400 \end{array}$$
Step 2-1: Calculate the test value $Tv_{i}$ of the credibility degree of $m_{i}$ as:
  $$\begin{array}{cc} \; & Tv_{1}=0.0782\;\;Tv_{2}=1.0429\\ \; & Tv_{3}=-0.1409\;Tv_{4}=-0.1793\\ \; & Tv_{5}=-0.1667 \end{array}$$
Step 2-2: Modify the adjacency matrix *A* to $A^{\prime }$:
  $$A^{\prime }=\left(\begin{array}{ccccc} 0 & 0 & 1 & 1 & 1\\ 0 & 0 & 0 & 0 & 0\\ 1 & 0 & 0 & 1 & 1\\ 1 & 0 & 1 & 0 & 1\\ 1 & 0 & 1 & 1 & 0 \end{array}\right)$$
Step 2-3: Generate the interaction graph $G^{\prime }$ between evidence as Figure 2.
Step 3-1: Fuse the connected evidence via the Dempster’s rule of combination; the process evidence $\underline{m_{i}}$ is computed. Since the connection graph is modified by a full connected graph, the process evidence generated by the proposed method is also the same. The process evidence $\underline{m_{1}}$ is shown as follows:
  $$\begin{array}{cc} \; & \underline{m_{1}}\left(A\right)=0.9649\\ \; & \underline{m_{1}}\left(B\right)=0.0006\\ \; & \underline{m_{1}}\left(C\right)=0.0344\\ \; & \underline{m_{1}}\left(A,C\right)=0.0000 \end{array}$$
Step 3-2: Fuse the process evidence via Dempster’s combination rule; the fusion results are shown in Table 2.

The more precise results of the proposed method are exhibited bellow.

$$\begin{array}{cc} \; & m(A)=0.999998\\ \; & m\left(B\right)=1.866277\times 10^{-13}\\ \; & m\left(C\right)=1.623702\times 10^{-6}\\ \; & m\left(A,C\right)=2.134348\times 10^{-33}\\ \; & m\left(A,B,C\right)=4.299348\times 10^{-17} \end{array}$$

- Discussion

From Case 1, we notice that $m_{2}$ is the highly conflicting evidence with others. The fusing results obtained by different combination methods are presented in Table 2. As shown in Table 2, except Dempster’s combination rule [13], Dubois and Prade’s method [24], PCR6 method [26], Murphy’s method [28], Deng et al.’s method [29], Yuan et al.’s method [30], Xiao’s method [33] and the proposed method all identify the correct target. Among the results shown in Table 2, after fusion by two rounds, the proposed method has the closest support to 1for target *A*, which demonstrates the proposed method is effective when conflicting evidence exists. In fact, by excluding the untrustworthy evidence from the connection graph, the adverse effects are greatly eliminated, so that the fusion of reliable evidence is reinforced from the other side.

### 5.2. Case2

- The decision-making application

In this subsection, an application case from [39] is considered, which was evaluated through a series of experiments implemented in the Internet of Things (IoT) and smart building projects by the CERIST-ALGERIA research center laboratory. In this scenario, 4 sensors are installed to monitor ambient light to optimize electrical lighting and energy control, and 4 hypotheses are defined as follows. $H_{1}$: The office is occupied and the lighting value exceeds 580 lx; $H_{2}$: The office is idle and the lighting value exceeds 580 lx; $H_{3}$: The office is occupied and the lighting value does not exceed 580 lx; and $H_{4}$: The office is empty and the lighting value does not exceed 580 lx. Ten percent of belief was assigned to θ to estimate the impact of the environment on evidence generation. The BBAs collected by four sensors are shown in Table 3.

- The fusion approach

**Figure 3:**
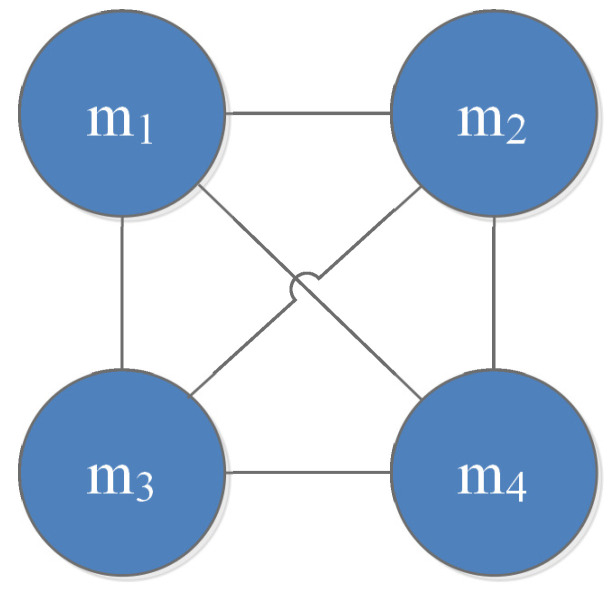
The interaction graph generated by the proposed method.

Step 1-1: Construct the divergence measure matrix $DMM={\left(d_{ij}\right)}_{k\times k}$ as follows:
  $$DMM=\left(\begin{array}{cccc} 0 & 0.0973 & 0.0909 & 0.0948\\ 0.0973 & 0 & 0.1046 & 0.1217\\ 0.0909 & 0.1046 & 0 & 0.0201\\ 0.0948 & 0.1217 & 0.0201 & 0 \end{array}\right)$$
Step 1-2: Obtain the average evidence distance $AD_{i}$ of $m_{i}$ as:
  $$\begin{array}{cc} \; & AD_{1}=0.0944\;AD_{2}=0.1079\\ \; & AD_{3}=0.0719\;AD_{4}=0.0789 \end{array}$$
Step 1-3: Calculate the support degree of $m_{i}$ as:
  $$\begin{array}{cc} \; & Sup_{1}=10.5982\;Sup_{2}=9.2687\\ \; & Sup_{3}=13.9153\;Sup_{4}=12.6745 \end{array}$$
Step 1-4: Compute the credibility degree of $m_{i}$ as:
  $$\begin{array}{cc} \; & Crd_{1}=0.2281\;Crd_{2}=0.1995\\ \; & Crd_{3}=0.2995\;Crd_{4}=0.2728 \end{array}$$
Step 2-1: Calculate the test value $Tv_{i}$ of the credibility degree of $m_{i}$ as:
  $$\begin{array}{cc} \; & Tv_{1}=0.0959\;\;Tv_{2}=0.2531\\ \; & Tv_{3}=-0.1654\;Tv_{4}=-0.0837 \end{array}$$
Step 2-2: Modify the adjacency matrix *A* to $A^{\prime }$:
  $$A^{\prime }=\left(\begin{array}{cccc} 0 & 1 & 1 & 1\\ 1 & 0 & 1 & 1\\ 1 & 1 & 0 & 1\\ 1 & 1 & 1 & 0 \end{array}\right)$$
Step 2-3: Generate the interaction graph $G^{\prime }$ between evidence as Figure 3.
Step 3-1: Fuse the connected evidence via the Dempster’s rule of combination; the process evidence $\underline{m_{i}}$ is computed. Since the connection graph is modified by a fully connected graph, the process evidence generated by the proposed method is also the same. The process evidence $\underline{m_{1}}$ is shown as follows:
  $$\begin{array}{cc} \; & \underline{m_{1}}\left(H_{1}\right)=0.9919\\ \; & \underline{m_{1}}\left(H_{2}\right)=0.0026\\ \; & \underline{m_{1}}\left(H_{3}\right)=0.0051\\ \; & \underline{m_{1}}\left(H_{4}\right)=0.0001 \end{array}$$
Step 3-2: Fuse the process evidence via Dempster’s combination rule; the fusion results are shown in Table 4.

The more precise results of the proposed method are exhibited bellow.

$$\begin{array}{cc} \; & m(H_{1})=0.999999\\ \; & m\left(H_{2}\right)=6.923081\times 10^{-11}\\ \; & m\left(H_{3}\right)=8.412098\times 10^{-10}\\ \; & m\left(H_{4}\right)=2.165541\times 10^{-14}\\ \; & m\left(\theta \right)=4.952586\times 10^{-15} \end{array}$$

- Discussion

From case 2, we notice that there is low conflict between the evidence. As shown in Table 4, in this usual case without strong conflicts, all methods identify the correct target. Additionally, the proposed method has the highest support for target $H_{1}$.

## 6. Conclusions

In this paper, a networked method to handle information fusion problems was proposed by taking the evidence interaction graph and DST into account. The original purpose is twofold. One is to reduce the impact of conflicting evidence on fusion, and the other is to realize information fusion in the way of multi-agent systems achieving consensus. However, even if the communication topology is established, the difference between evidence and the relative neighboring error between multi-agents is not the same, and the fusion between evidence and the collaborative control of multi-agent systems cannot be carried out in the same way. As a result, only the first goal was achieved, i.e., the influence of conflicting evidence generated by various reasons on fusion was reduced, but the collaborative method between evidence still needs to be explored. In summary, the proposed fusion method is an attempt to bring information fusion into a part of networked systems, which provides a new inspiration for information fusion and networked systems. However, while the fusion performance is satisfactory, the amount of calculation is also significantly increased. In the future, we intend to develop some new rules for modifying evidence connections. In particular, by reducing the number of connected edges or making the connection weights of the edges more refined, we will try to achieve the purpose of reducing computation.

## Figures and Tables

**Table 1:** The basic belief assignments for multi-sensor-based target recognition. (Reprinted from [33] Copyright (2022), with permission from Elsevier).

BBA	***A***	***B***	***C***	A,C
$m_{1}$	0.41	0.29	0.30	0.00
$m_{2}$	0.00	0.90	0.10	0.00
$m_{3}$	0.58	0.07	0.00	0.35
$m_{4}$	0.55	0.10	0.00	0.35
$m_{5}$	0.60	0.10	0.00	0.30

**Table 2:** Combination results of the evidence in terms of different combination rules.

Method	***A***	***B***	***C***	A,C	Target
Dempster [13]	0.0000	0.1422	0.8578	0.0000	*C*
Dubois and Prade [24]	0.7504	0.0160	0.0158	0.0832	*A*
PCR6 [26]	0.4518	0.3624	0.0438	0.1420	*A*
Murphy [28]	0.9620	0.0210	0.0138	0.0032	*A*
Deng et al. [29]	0.9820	0.0039	0.0107	0.0034	*A*
Yuan et al. [30]	0.9886	0.0002	0.0072	0.0039	*A*
Xiao [33]	0.9905	0.0002	0.0061	0.0043	*A*
Proposed method	1.0000	0.0000	0.0000	0.0000	*A*

**Table 3:** The basic belief assignments for IoT decision making. (Reprinted from [39] Copyright (2022), with permission from Elsevier).

BBA	$H_{1}$	$H_{2}$	$H_{3}$	$H_{4}$	θ
$m_{1}$	0.648	0.153	0.090	0.009	0.100
$m_{2}$	0.621	0.072	0.198	0.009	0.100
$m_{3}$	0.729	0.054	0.099	0.018	0.100
$m_{4}$	0.747	0.063	0.081	0.009	0.100

**Table 4:** Combination results of the evidence for case 2.

BBA	$H_{1}$	$H_{2}$	$H_{3}$	$H_{4}$	Target
Dempster [13]	0.9918	0.0027	0.0051	0.0001	$H_{1}$
Dubious and Prade [24]	0.7704	0.0110	0.0200	0.0003	$H_{1}$
PCR6 [26]	0.9158	0.0246	0.0428	0.0005	$H_{1}$
Xiao [33]	0.9919	0.0026	0.0051	0.0001	$H_{1}$
Jiang et al. [40]	0.9908	0.0030	0.0058	0.0001	$H_{1}$
Wang et al. [39]	0.9921	0.0025	0.0050	0.0001	$H_{1}$
Proposed method	1.0000	0.0000	0.0000	0.0000	$H_{1}$

## Data Availability

Not applicable.
